# Differential gene expression in response to water deficit in leaf and root tissues of soybean genotypes with contrasting tolerance profiles

**DOI:** 10.1590/1678-4685-GMB-2018-0290

**Published:** 2020-05-29

**Authors:** Rafaela Ribeiro Reis, Liliane Marcia Mertz-Henning, Juliana Marcolino-Gomes, Fabiana Aparecida Rodrigues, Silvana Rockenbach-Marin, Renata Fuganti-Pagliarini, Alessandra Koltun, Leandro Simões Azeredo Gonçalves, Alexandre Lima Nepomuceno

**Affiliations:** 1 Universidade Estadual de Londrina, Departamento de Biologia e Departamento de Agronomia, Londrina, PR, Brazil; 2 Embrapa Soybean, Empresa Brasileira de Pesquisa Agropecuária, Londrina, PR, Brazil; 3 Conselho Nacional de Desenvolvimento Científico e Tecnológico - CNPq, Brasília, DF, Brazil; 4 Instituto Federal de Educação, Ciência e Tecnologia de Mato Grosso, Coxim, MS, Brazil; 5 Universidade Estadual de Maringá, Maringá, PR, Brazil

**Keywords:** Transcription factors, osmoregulation, AP2/EREBP, WRKY

## Abstract

Water deficit is one of the major limitations to soybean production worldwide, yet the genetic basis of drought-responsive mechanisms in crops remains poorly understood. In order to study the gene expression patterns in leaves and roots of soybean, two contrasting genotypes, Embrapa 48 (drought-tolerant) and BR 16 (drought-sensitive), were evaluated under moderate and severe water deficit. Transcription factors from the *AP2/EREBP* and *WRKY* families were investigated. Embrapa 48 showed 770 more up-regulated genes than BR 16, in eight categories. In general, leaves presented more differentially expressed genes (DEGs) than roots. Embrapa 48 responded to water deficit faster than BR 16, presenting a greater number of DEGs since the first signs of drought. Embrapa 48 exhibited initial modulation of genes associated with stress, while maintaining the level of the ones related to basic functions. The genes expressed exclusively in the drought-tolerant cultivar, belonging to the category of dehydration responsive genes, and the ones with a contrasting expression pattern between the genotypes are examples of important candidates to confer tolerance to plants. Finally, this study identified genes of the *AP2/EREBP* and *WRKY* families related to drought tolerance.

## Introduction

Soybean, considered a worldwide commodity, is highly affected by biotic and abiotic stresses. In this context, climate changes and expansion of agricultural areas have established new requirements for crop cultivation and productivity (Haggag *et al.*, 2015). Recent data show that water deficit stress has resulted in strong soybean yield losses globally, emphasizing that drought is one of the most stressful environmental factors to economic crops. In Brazil, the second largest producer in the world and one of the few countries that could considerably increase its soybean production in the next decades, water deficit hinders the full exploitation of the country's potential. From the cropping seasons of 2003/2004 to 2014/2015, losses due to drought events were estimated to be about US$ 46.6 billion (Fuganti-Pagliarini *et al.*, 2017).

When subjected to environmental stress conditions, such as water deficit, plants trigger molecular mechanisms of prevention and protection to avoid cell damages. Genes involved in drought responses can be classified into two groups: functional and regulatory (De Carvalho, 2008; Molina *et al.*, 2008). Different stress response strategies involving functional genes, which act directly against the damage caused by stress, are activated to circumvent the effects of adverse conditions. These responses include morphological alterations in leaves, shoots and roots, variations in gene regulation, which triggers innumerable cascade expression inductions, changes in stomatal conductance mediated by abscisic acid (ABA), osmoregulation, and the activity of antioxidant enzymes (Nakashima *et al.*, 2014). The precise control of the metabolic pathways and regulatory network of events that are triggered by stress is key to determining the tolerance level of the plant. In this context, transcription factors (TFs) play a crucial role in the regulation of the process of signaling the perception of stress and transmitting it to the transduction pathway, activating defense genes. Regulatory genes are composed of several classes and families, and are involved in the activation/inactivation of genes related to a great number of biological processes (Zhu, 2016). In *A. thaliana* nearly 6% of the proteome is dedicated to TFs (Rayko *et al.*, 2010).

Among the TFs that have been linked to abiotic stress responses is the *AP2/EREBP* family, which includes a large group of plant-specific factors and is characterized by the presence of a highly conserved AP2/ERF (APETALA2/Ethylene Responsive Factor) binding domain, consisting of 58-60 amino acids. These TFs interact directly with GCC-box and/or DRE/CRT (Dehydration-responsive element/C-repeat element) cis-acting elements in the promotor region (Yamaguchi-Shinozaki and Shinozaki, 2006).

*WRKY* also represents a transcription factor family with multiple roles in biotic/abiotic stress responses, as well as in developmental/physiological processes (Jiang *et al.*, 2015). It is considered the most important TF family in plants and consists of ? 60 amino acid, four-stranded β-sheet WRKY DNA binding domains/ (DSD and Zing-finger) motifs. WRKY TFs interact with W-box (with the core motif TTGACC/T) and with clustered W-boxes present in the promoter region of of downstream genes. Recently, studies of Chu *et al.* (2015) demonstrated that the overexpression of the *GhWRKY41* gene enhanced salt tolerance and Li *et al.* (2015) observed that the overexpression of the *SpWRKY1* gene boosted drought tolerance, by studying *reactive oxygen species (ROS)* levels and stomatal conductance regulation in transgenic tobacco.

To identify crucial components that confer tolerance to plants, several transcriptomic analyses of plants subjected to drought have previously been performed in many species under different experimental conditions. In soybean, a research was conducted using RNA sequencing (RNA-seq) as a platform for evaluating gene expression (Rodrigues *et al.*, 2015), in which daytime transcriptome fluctuations were observed during water deficit stress. Furthermore, the characterization of the expression profile of a transcription factor from the family GmAP2/EREBP was carried out (Marcolino-Gomes *et al.*, 2015). A similar study employed Suppressive Subtractive Hybridization (SSH) for transcriptome analysis in soybean under drought conditions and found some differentially expressed genes and modulation of gene classes in both cultivars evaluated (Embrapa 48 and BR 16) (Rodrigues *et al.*, 2012).

Although studies of RNA-seq with soybean under water deficit have already been conducted, a more complete analysis that integrates the evaluation of different genotypes with distinct tolerance profiles, in different tissues and stress levels, has not yet been performed.

Therefore, in order to expand the understanding of and reveal new perspectives about the oscillation of the soybean transcriptome under water deficit conditions, this study compared the gene expression profiles of contrasting soybean genotypes (Embrapa 48 and BR 16) using RNA-seq, enabling an evaluation of the main molecular peculiarities that differentiate these cultivars in leaf and root tissues under drought (moderate and severe levels). Additionally, this study aimed at gaining insight into the dynamics of some TFs of the *AP2/EREBP* and *WRKY* families as a response to water deficit stress in the contrasting soybean genotypes.

## Material and Methods

### Plant material and experimental design

Transcriptomic data from soybean cultivated under water deficit conditions in a hydroponic system were evaluated based on the method and experimental design described by Martins *et al.* (2008). Seeds of the soybean cultivars Embrapa 48 and BR 16, classified as tolerant and sensitive to water deficit, respectively (Oya *et al.*, 2004), were germinated on germination paper during four days in a growth chamber at 25 ± 1 ºC and 100% relative humidity (RH). Seedlings were placed in 36 1-L boxes containing Hoagland's solution (50%, without modifications) (Hoagland and Arnon, 1950), which was continuously aerated and replaced on a weekly basis. The boxes were then transferred to a greenhouse with a natural photoperiod of approximately 12/12 h light/dark cycle, temperature of 30 ± 5 ºC and RH of 60 ± 10%, where the plants grew until the V4 stage (Fehr *et al.*, 1971). The experimental design was randomized complete block, where treatments were arranged in a factorial scheme 2 × 5, with three replicates, each one comprised of 5 plants. The factors were two cultivars (Embrapa 48 and BR 16) and five treatment sampling times (0, 25, 50, 125 and 150 min under water deficit). The stress was applied by removing the plants from the hydroponic solution and leaving them in boxes without nutrient solution for up to 150 min under ambient conditions. For each sampling time, leaves and roots from 15 plants (5 plants for each replicate) were collected, pooled and frozen in liquid nitrogen before storage at −80 ºC.

### Library construction and sequencing run

Total RNA from leaf and root samples from Embrapa 48 and BR 16 was extracted using the TRIzol reagent (Invitrogen). Bulk samples of total RNA were made by pooling samples of the same tissue to generate the moderate stress library (25 and 50 min), the severe stress library (125 and 150 min) and the control (0 min, not stressed). After DNase treatment (Life Technologies Grand Island, NY, USA), high-quality total RNA was used to analyze the transcripts. The RNA-seq libraries were built using the Nugen-Ovation kit according to the manufacturer's instructions (NuGEN Technologies Inc., San Carlos, CA, USA). In total, 12 libraries (2 genotypes x 3 stress periods including the control treatment x 2 tissues) were sequenced. The libraries obtained were subjected to sequencing by an Illumina HiSeq2000 system (Illumina, San Diego, CA, USA). Data corresponding to this manuscript are deposited in the NCBI Sequence Read Archive (SRA) database (accession: PRJNA615913).

### Mapping of reads and functional classification

Thousands of reads were generated in each library. Mapping of reads was performed with the soybean genome (Phytozome Glycine max v1.1) using the GeneSifter platform (http://www.geospiza.com/Products/AnalysisEdition.shtml). To compare gene expression between different treatment sampling times (stress level), log 2 was used to perform the normalization in Reads per Mapped Million (RPM). A *t-*test analysis was conducted to compare data from the two groups generated (moderate and severe stress level). Contig sequences were submitted to the NCBI non-redundant protein database through BlastX (Altschul et al*.*, 1997) to search for similarity with known proteins. In addition, sequences were analyzed by the software AutoFact (Koski *et al.*, 2005), which is an automated annotation tool that assigns biological information for a given sequence by comparing different databases. We used the UniRef90, UniRef100, KEGG (Kanehisa and Goto, 2000), Pfam (Finn *et al.*, 2010) and Smart (Schultz *et al.*, 1998) databases. Additionally, to establish the Gene Ontology (GO) terms (Ashburner et al*.*, 2000) we employed the Blast2Go program to classify the sequences according to the molecular function and the biological process described for the respective proteins (Götz *et al.*, 2008; Carbon *et al.*, 2009).

### Analysis of differential gene expression

Using the GeneSifter platform (http://www.geospiza.com/Products/AnalysisEdition.shtml), we applied a pairwise comparison between the control (0 min) and water deficit treatments (moderate and severe levels). In the pairwise analysis, a ratio of expression (fold-change: fc) was generated by dividing values of gene expression under water deficit levels and the control condition. The statistical significance of DEGs (differentially expressed genes) was obtained by using the Bioconductor package edgeR (Robinson *et al.*, 2010), corrected by the Benjamini and Hochberg method (Benjamini and Hochberg, 1995), which calculates the False Discovery Rate (FDR) avoiding inflation of type-1 error. We only considered DEGs genes that showed fold-change ≥ 2 (up), ≤ −2 (down) and presented more than 20 mapped reads (RPM ≥ 9) in at least one of the libraries. We also applied a stringent statistical significance cutoff (adjusted *p*-value ≤ 0.01) to improve confidence.

### Classification and analysis of functional gene categories

Analysis using MapMan 3.6 ORC1 (http://mapman.gabipd.org/web/guest/mapman-version-3.6.0) allowed the detection of differentially expressed genes, which were calculated based on a calibration at time 0 of stress (control), and were classified by functional categories in several pathways. Twelve categories were mapped and selected for analysis: transcription factor, amino acid, tricarboxylic acid (TCA) cycle, sucrose, photorespiration, light reactions, lipids, cell wall, abscisic acid (ABA) precursor, ABA metabolism, drought/salt, peroxidase and development. Within the transcription factor category, genes from two groups were further analyzed: the *AP2/EREBP* and *WRKY* families.

### Validation of gene expression

#### Gene selection, primer design and efficiency analysis

Seven genes were selected for real-time PCR analysis aimed at validating the results obtained in the RNA-seq libraries: Glyma17g17860 (*LEA18*), Glyma08g01430 (*WRKY75*), Glyma05g32040 (*AP2*), Glyma0041s00200 (*AP2*), Glyma13g17250 (*ERF018*), Glyma17g14110 (*DREB1E*) and Glyma20g29410 (*DREB1A*) (Table S1). Glyma17g17860 (*LEA18*), which plays a crucial role in cellular dehydration tolerance, was investigated to confirm that the water deficit stress treatment was successfully applied. The other genes were selected as they belong to relevant TF families, *AP2* and *WRKY*, and due to their discrepant differential expression between the tolerant and sensitive cultivars, suggesting they play an important role in the drought resistance mechanism. Primers for the target genes were designed based on the GeneModels using the program Primer Express 3.0 (Applied Biosystems/Life Technologies, Grand Island, NY, USA) (Table S1). Primers were determined for the 3'end of each gene, and the amplicons spanned up to 150 base pairs (bp). The primer sequences were then BLASTed against the soybean genome (Phytozome database v1.0, http://www.phytozome.net/search.php) to verify their specificity. Additionally, standard curves were produced from serial dilutions of a cDNA pool to estimate the efficiency of the PCR amplification reactions.

#### RT-qPCR analysis

Relative expression levels of the target genes were measured in root and leaf samples from Embrapa 48 and BR 16 plants for each bulk time point (0, 25–50 min, 125–150 min under water deficit) and level of stress (control, moderate and severe), using three biological replicates with technical triplicates. After DNAse treatment (Invitrogen/Life Technologies, Grand Island, NY, USA), high quality total RNA was used to synthesize cDNA strands (Superscript II First Strand Synthesis, Invitrogen/Life Technologies, Grand Island, NY, USA), and cDNA quality was verified using a standard PCR reaction with an actin primer that spanned an intronic region. The genes were then amplified by a StepOne RT-qPCR Thermocycler (Applied Biosystems/Life Technologies, Grand Island, NY, USA) with the following cycling FAST parameters: 95 ºC for 20 sec, 40 cycles at 95 ºC for 3 sec, 60 ºC for 30 s, then melt curve (95 ºC for 15 s and 60 ºC for 1 min). Data were collected during the extension phase, and dissociation curves were performed by heating each amplicon from 60 to 95 ºC and taking readings at one-degree intervals to verify the specificity of the primers. The Rest2009 software package (Pfaffl *et al.*, 2002) was used to evaluate the data, providing a robust statistical analysis. The RT-qPCR was normalized by taking the geometric average of the selected endogenous genes [*FYVE* and *B-actin*, described by Marcolino-Gomes *et al.* (2015)], and the control plants (0 min under stress) were used to calibrate the relative expression. Hypothesis testing was used to determine whether the differences between the control and treatment conditions were significant (Pfaffl *et al.*, 2002).

## Results

### Comparative analysis of deferentially expressed genes in leaf and root samples in moderate and severe stress libraries

Transcriptomic analysis by RNA-seq platform was used to identify differentially expressed genes in two soybean cultivars (Embrapa 48 and BR 16) under water deficit conditions. Root and leaf samples were extracted from plants subjected to moderate stress (25 and 50 min under water deficit) and severe stress (100 and 150 min under water deficit). The differential expression was calculated based on a calibration using control samples (not stressed- 0 min). A total of 47,177,642 reads were obtained after adapter removal, which were then mapped and only sequences with a maximum of two mismatches in the first 32 bases were selected, reducing the reads to 23,246,624 (Table 1). Furthermore, 55,787 annotated mRNAs were obtained and from this total 51,322 were hit by at least one mRNA and 39,951 passed the low count filter.

**Table 1:** Differential gene expression in leaves and roots from the cultivars BR 16 and Embrapa 48 under conditions of moderate and severe water deficit stress.

Sample	Reads after adapter removal	Mapped reads	% of mapped reads (mean)
Embrapa 48 leaf	12,480,047	6,139,313	49.2
Embrapa 48 root	12,241,551	6,137,155	50.2
BR 16 leaf	11,305,266	5,414,745	47.6
BR 16 root	11,150,778	5,555,411	49.7
Total	47,177,642	23,246,624	-

Differentially expressed genes under water deficit conditions were distributed between up- and down-regulated (Table 2) and it can be inferred that the stress responses were more evident in leaves, where DEGs responded more intensively compared to roots for Embrapa 48. Regarding leaves of plants subjected to moderate stress, Embrapa 48 showed more up-regulated genes, in contrast to BR 16 (Table 2) which presented a greater number of down-regulated genes (Table 2). Moreover, when the total number of DEGs (up- and down-regulated) were considered, Embrapa 48 had about 11% more DEGs than BR 16, with a total of 25203, whereas the sensitive cultivar had only 22,517 (Table 2).

**Table 2:** General information obtained in the RNA-seq analysis of the cultivars Embrapa 48 and BR 16 under water deficit conditions.

TREATMENT	Up-regulated genes number		Down-regulated genes number	
	Embrapa 48	BR 16	Embrapa 48	BR 16
Leaf (moderate stress)	4,693	2,009	1,238	2,128
Leaf (severe stress)	4,247	2,311	2,694	5,167
Root (moderate stress)	2,479	2,274	3,162	2,165
Root (severe stress)	3,031	2,834	3,659	3,629
TOTAL	14,450	9,428	10,753	13,089
Embrapa 48 total genes	25,203			
BR 16 total genes	22,517			

**Figure 1:**
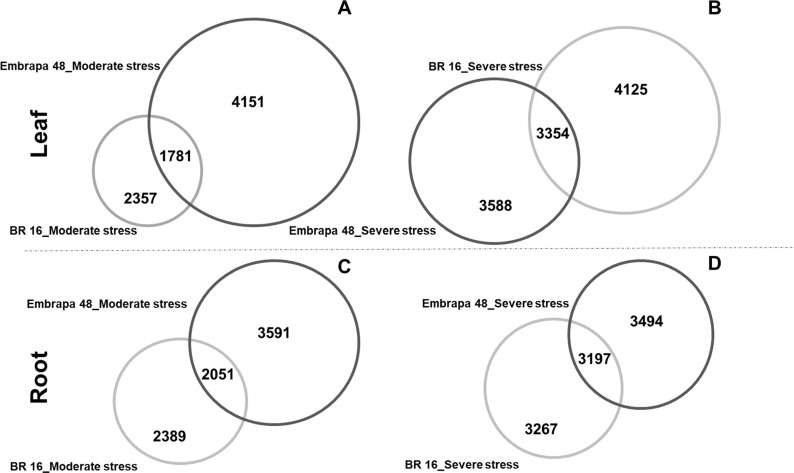
Venn diagram. Number of genes differentially expressed in both libraries (moderate and severe water defict stress), highlighting common genes between cultivars. (A) Leaf from BR 16 and Embrapa 48 in moderate stress library; (B) leaf from BR 16 and Embrapa 48 in severe stress library; (C) root from BR 16 and Embrapa 48 in moderate stress library; (D) root from BR 16 and Embrapa 48 in severe stress library. The size of the circle represents the number of genes. Dark gray circles represent the cultivar Embrapa 48 and light gray circles refer to the cultivar BR 16.

In general, Embrapa 48 presented more differentially expressed genes than BR 16 (Figure 1A, C and D) for almost all treatments. However, analyzing leaves from the severe stress library, it was possible to observe a great number of exclusive DEGs (4,125) for BR 16, while Embrapa 48 presented a slightly lower number (3,588) (Figure 1B). Considering roots, BR 16 had 2,389 exclusive genes induced under the moderate stress and 3,267 under the severe level. This pattern was not observed for Embrapa 48. For this genotype the number of DEGs showed no significant change from one level of stress to the other (3,591 with moderate stress and 3,494 with severe stress) (Figure 1C and D). In general, BR 16 showed higher gene activation with severe stress, almost doubling the number of DEGs when compared to the moderate stress (Figure 1). In contrast, Embrapa 48 demonstrated a more active gene modulation for the moderate stress library (Figure 1A and C).

**Figure 2:**
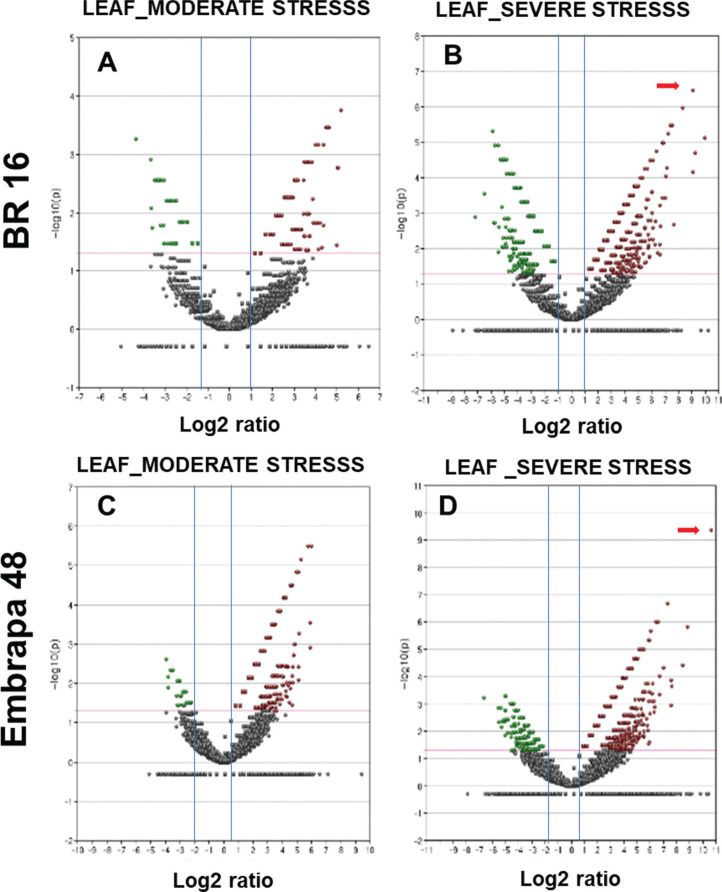
Volcano plots. Expression data (fold-change) were plotted using log2 scale (x-axis) and -log_10_ transformation of the *p*-value (y-axis). Datasets were filtered to remove genes with low expression levels (blue line on the x-axis ≥1 and −1 and red lines on the y-axis); the red line is the threshold applied to delimit a significance cut off (*p* < 0.01). The red points were used to represent up-regulated genes and green points down-regulated genes. The red arrows indicate the highest expression of the same genes for (A) leaf from BR 16 in moderate stress library; (B) leaf from BR 16 in stress severe stress library; (C) leaf from Embrapa 48 in moderate stress library; (D) leaf from Embrapa 48 in severe stress library.

Among the genes that presented the highest differential expression, revealed by RNA-seq (Figure 2), Glyma09g31740 (Dehydrin) stood out. This gene was identified in leaves of Embrapa 48 under severe stress and presented the highest expression level among all up-regulated genes with a 1594.81-fold change (Log 2 = 10.63; indicated by a red arrow in Figure 2D). Whereas, in leaves of BR 16 subjected to severe stress, Glyma20g29770 (no annotation) reached a high level of expression, with a fold change of 1186.29 (Log 2 = 10.21; indicated by red arrow in Figure 2B).

Concerning roots in the moderate library, Embrapa 48 showed more down-regulated genes than BR 16 (Table 2). For the severe library, the difference between cultivars in the general expression profile was not as evident; both cultivars showed a high number of DEGs (Table 2).

**Figure 3:**
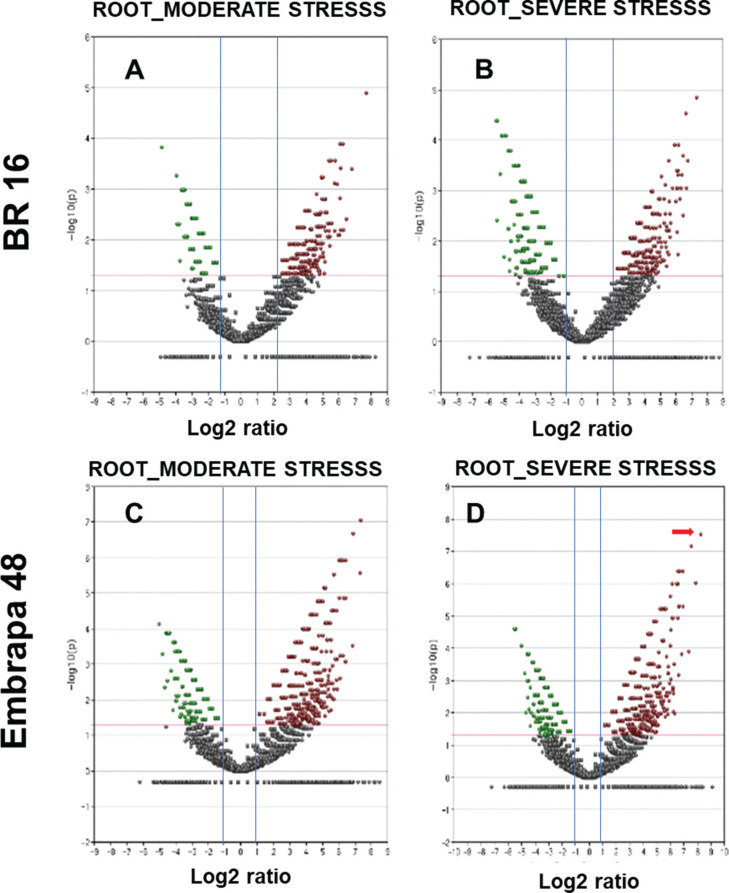
Volcano plots. Expression data (fold-change) were plotted using log2 scale (x-axis) and -log_10_ transformation of the *p*-value (y-axis). Datasets were filtered to remove genes with low expression levels (blue line on the x-axis ≥1 and −1 and red lines on the y-axis); the red line is the threshold applied to delimit a significance cut off (*p* < 0.01). The red points were used to represent up-regulated genes and green points down-regulated genes. The red arrows indicate the highest expression of the same genes for (A) roots from BR 16 in moderate stress library; (B) roots from BR 16 in severe stress library); (C) roots from Embrapa 48 in moderate stress library); (D) roots from Embrapa 48 in severe stress library.

In Figure 3, volcano plots show up- and down-regulated genes in Embrapa 48 and BR 16 root tissues from the moderate and severe stress libraries. The gene Glyma03g26310 (*AP2* domain) was highlighted in Embrapa 48 subjected to severe stress, with a high fold-change (544.18; Log 2 = 9.08), when compared to the other genes within the same treatment (indicated by red arrow in Figure 3D).

**Figure 4:**
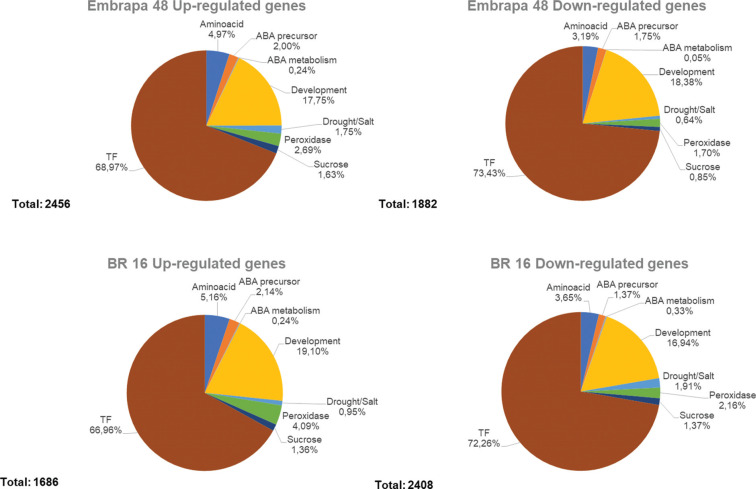
Graphic representation of eight gene categories (enriched biological process from MapMan 3.6 ORC), analyzed in up- and down-regulated gene groups from the cultivars BR 16 and Embrapa 48.

When leaf and root samples were analyzed for eight specific categories, Embrapa 48 had 770 more up-regulated genes than BR 16 (from a total of 2,456 and 1,686 expressed genes, respectively), whereas down-regulated genes totaled 1,882 in Embrapa 48 and 2,408 in BR 16 (Figure 4).

### Analysis of gene expression patterns and functional roles in leaf under water deficit stress

**Figure 5:**
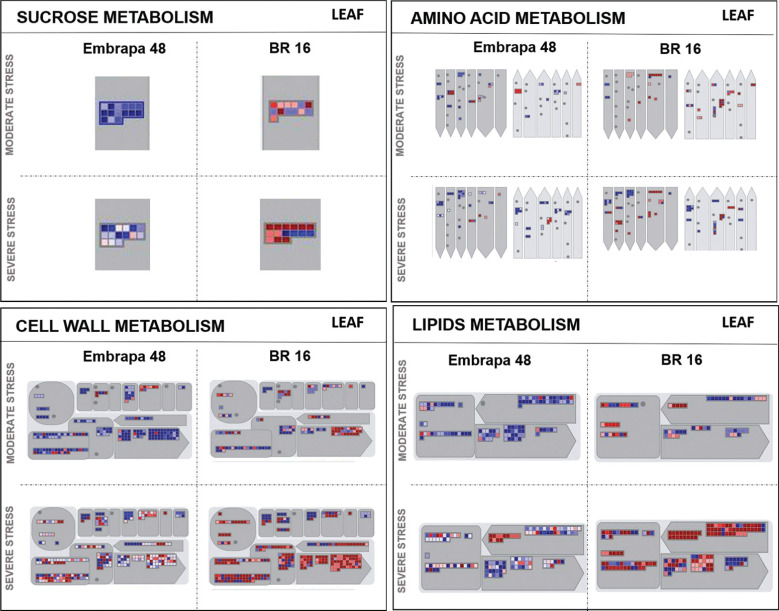
Metabolism overview mapping (MapMan 3.6 ORC). Genes that were differentially expressed in response to water deficit were mapped to specific stress-related gene groups (sucrose, amino acid, cell wall and lipid metabolism) for leaf from Embrapa 48 and BR 16 under moderate and severe stress. The color scale shows the log2 fold-change: red = down-regulated and blue = up-regulated.

**Figure 6:**
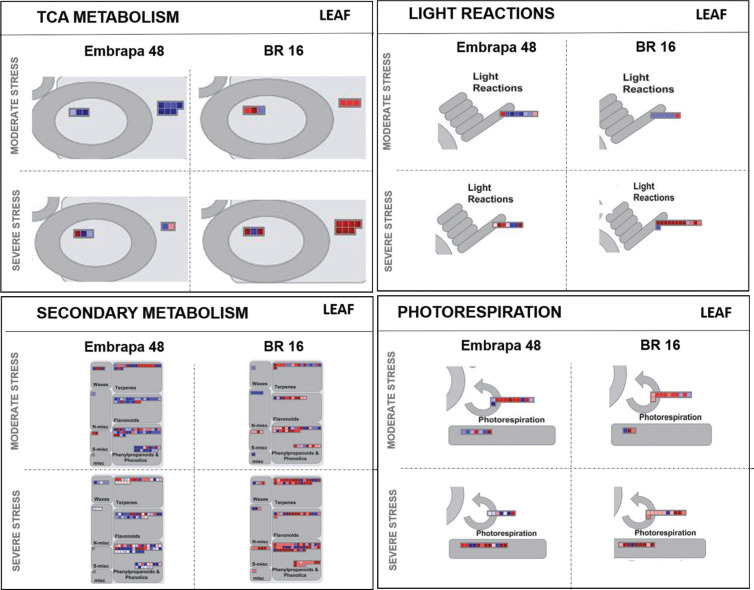
Metabolism overview mapping (MapMan 3.6 ORC). Genes that were differentially expressed in response to water deficit were mapped to specific stress-related gene groups (TCA and secondary metabolism, light reactions and photorespiration) for leaf from Embrapa 48 and BR 16 under moderate and severe stress. The color scale shows the log2 fold-change: red = down-regulated and blue = up-regulated.

RNA-seq transcriptome data of Embrapa 48 and BR 16 genotypes showed significant differential expression patterns for leaves, according to the type of metabolism and biological process (Figures 5 and 6). Twelve categories were selected based on the results of MapMan 3.6 ORC bioinformatics tool: transcript factor, amino acid, TCA cycle, sucrose, photorespiration, light reactions, lipids, cell wall, ABA precursor, ABA metabolism, drought/salt, peroxidase and development. From the data gathered, it is possible to observe the impact of water deficit stress, with the greatest difference between cultivars being detected in the leaf samples (Figures 5 and 6).

Considering the results obtained from leaf, in Embrapa 48 most of the genes presented an up-regulation pattern in all categories, indicating gene modulation against drought damage (Figures 5 and 6). Furthermore, the cells maintained the basic plant metabolism processes, such as photosynthesis, respiration, growth and development as well as the carbohydrate and nitrogen pathways and, thus, expressing both primary and secondary metabolism genes (Figures 5 and 6). A different profile was observed for BR 16, for instance, genes were strongly down-regulated in the moderate stress (Figures 5 and 6). When plants were subjected to a severe level of water deficit, the number of down-regulated genes increased for both cultivars, but with a higher intensity in the sensitive genotype BR 16 (Figures 5 and 6).

**Figure 7:**
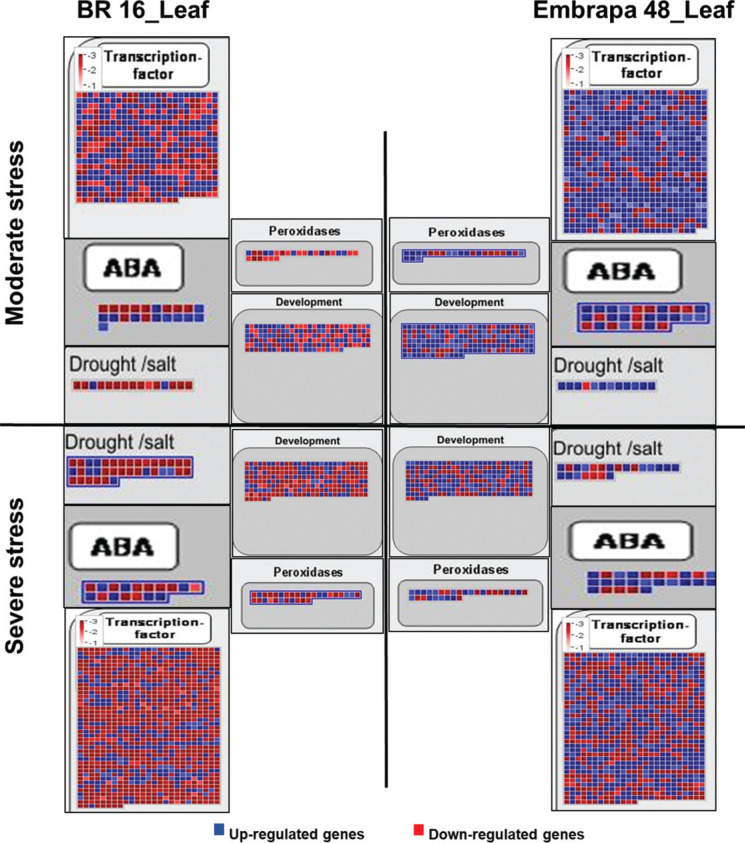
Modified maps of MapMan 3.6 ORC. Genes that were differentially expressed in response to water deficit were mapped to specific stress-related pathways for leaf from BR 16 and Embrapa 48 under moderate and severe stress. The color scale shows the log2 fold-change: red = down-regulated and blue = up-regulated.

For the functional category, which encompasses regulatory and defense genes such as transcription factors, ABA related and drought/salt responsive genes, the tolerant cultivar presented a higher number of up-regulated genes compared to the sensitive genotype for both levels of drought treatment (Figure 7). This is particularly true for the moderated stress.

Classes of genes responsive to drought were predominantly up-regulated in leaf samples from Embrapa 48 (25 genes) when compared to BR 16 (11 genes), for both libraries (moderate and severe) (Figure 7). Some genes belonging to this same class, such as Glyma09g40090 (dehydration-responsive protein-related genes), were differentially expressed (up-regulated) under moderate stress, however, they had a higher fold-change (Fc) in Embrapa 48 (5.3) compared to BR 16 (3.6). Additionally, with moderate stress, the genes Glyma14g06200, Glyma13g35970, Glyma18g53780, Glyma02g05840, Glyma16g32180, Glyma11g35590, Glyma01g07020, Glyma09g26650, and Glyma01g07020 were exclusively expressed in Embrapa 48.

Among all the DEGs in leaf samples from Embrapa 48 under moderate stress, Glyma03g04920 (Fc 34.36), Glyma13g22420 (Fc 22.26), Glyma10g03640 (Fc 21.02), Glyma11g13940 (Fc 19.32), Glyma13g43970 (Fc 18.55), Glyma01g26230 (Fc 17.75), Glyma12g05910 (Fc 16.96), Glyma15g05350 (Fc 15.9), Glyma17g07070 (Fc 15.66), and Glyma11g15060 (Fc 15.63) showed the highest levels of expression. As for leaf samples from Embrapa 48 under severe drought, Glyma03g29440 (Fc 192.2), Glyma04g00710 (Fc 63.66), Glyma07g38580 (Fc 60.31), Glyma11g05530 (Fc 56.55), Glyma17g11160 (Fc 43), Glyma16g04440 (Fc 37.74), Glyma11g21420 (Fc 36.85), Glyma08g18900 (Fc 35.51), Glyma07g06620 (Fc 34.34), and Glyma18g52700 (Fc 32.39) stood out among the DEGs identified in this group, showing the highest expression profiles.

Some genes are notable for being related to the contrasting water deficit response of Embrapa 48 (tolerant) and BR 16 (sensitive), and therefore, these genes possibly act in the drought tolerance. An example of a gene associated with moderate stress is Glyma13g35970, described as a dehydration-responsive protein RD22, which was highly expressed in Embrapa 48, reaching a fold-change of 31.8, whereas under severe drought, this gene presented a fold-change of 11.17 in Embrapa 48 and −6.02 in BR 16.

### Comparative analysis of gene expression patterns and functional roles in root under water deficit stress

**Figure 8:**
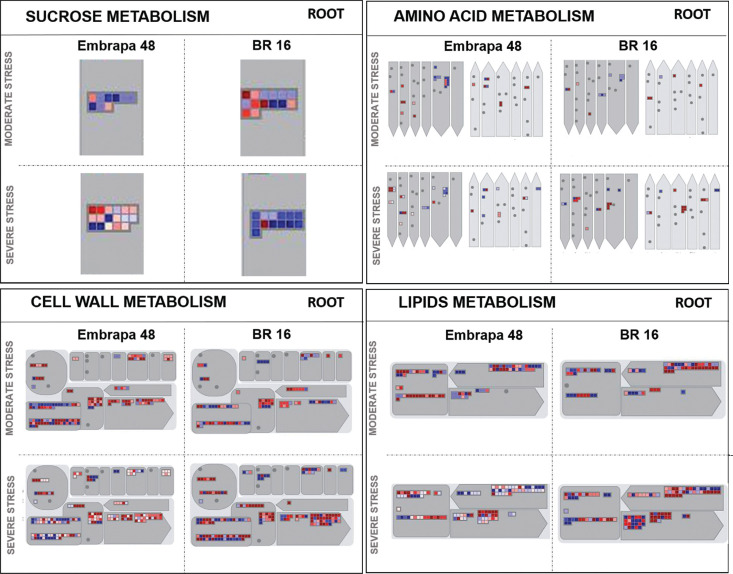
Metabolism overview mapping (MapMan 3.6 ORC). Genes that were differentially expressed in response to water deficit were mapped to specific stress-related gene groups (sucrose, amino acid, cell wall and lipid metabolism) for root from Embrapa 48 and BR 16 under moderate and severe stress. The color scale shows the log2 fold-change: red = down-regulated and blue = up-regulated.

**Figure 9:**
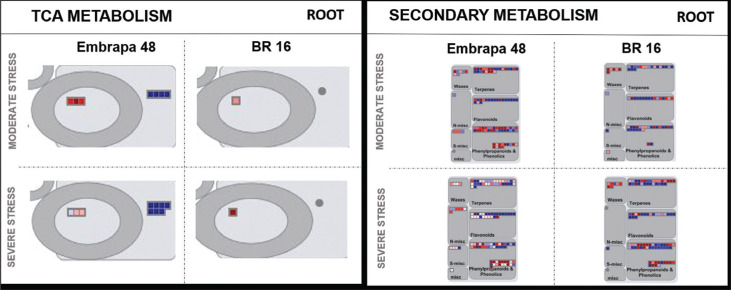
Metabolism overview mapping (MapMan 3.6 ORC). Genes that were differentially expressed in response to water deficit were mapped to specific stress-related gene groups (TCA and secondary metabolism). The color scale shows the log2 fold-change: red = down-regulated and blue = up-regulated.

**Figure 10:**
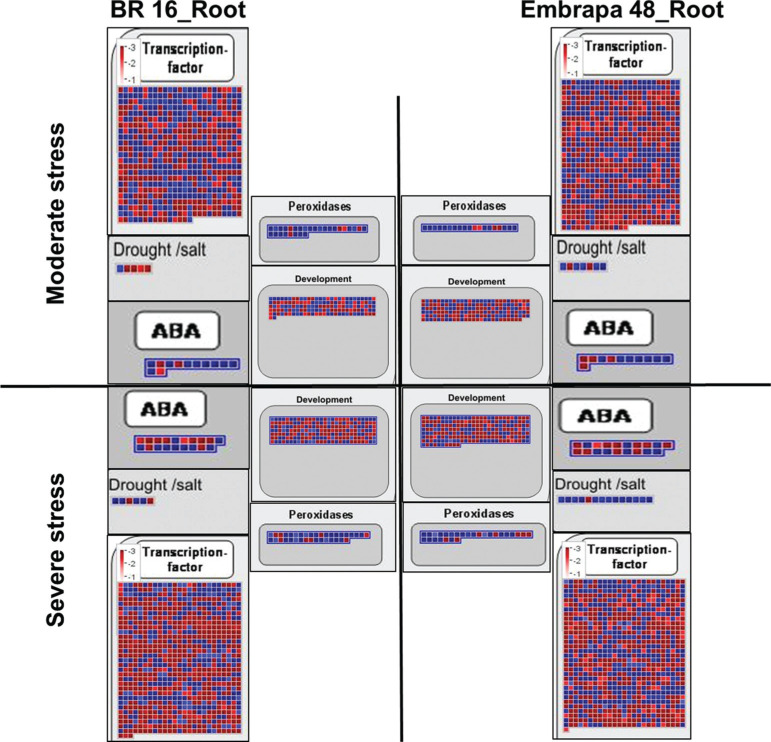
Modified maps of MapMan 3.6 ORC. Genes that were differentially expressed in response to water deficit were mapped to specific stress-related pathways for root from BR 16 and Embrapa 48 under moderate and severe stress. The color scale shows the log2 fold-change: red = down-regulated and blue = up-regulated.

Based on the results obtained from the MapMan 3.6 ORC1 bioinformatics tool, some differences may be emphasized in roots under the moderate stress, in which the tolerant cultivar presented most of the genes with an up-regulation pattern in sucrose and TCA categories (Figures 8 and 9). However, under severe stress, BR 16 genes related to sucrose were more up-regulated compared to Embrapa 48 (Figure 8). The genes from the amino acid category were not highly induced in roots (Figure 8). Regarding the functional category, specifically genes responsive to drought, in roots of Embrapa 48 they were up-regulated regardless of the level of water deficit (Figure 10). For both cultivars, a greater number of peroxidase and ABA genes was found in the severe stress library with predominance of up-regulation (Figure 10).

**Figure 11:**
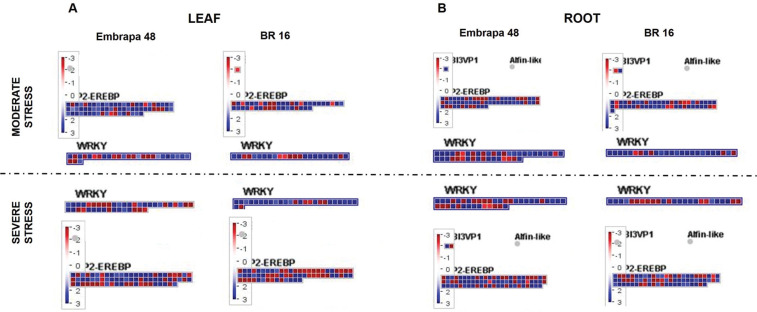
Modified maps of MapMan 3.6 ORC. Genes differentially expressed in response to water deficit were mapped to specific stress-related TF families AP2/EREBP and WRKY for (A) leaf from Embrapa 48 and BR 16 and (B) root from Embrapa 48 and BR 16 under moderate and severe stress. The color scale shows the log2 fold-change: red = down-regulated and blue = up-regulated.

Two important TF families related to biotic and abiotic stresses, *AP2/EREBP and WRKY*, were selected be further investigated. The expression of the *AP2/EREBP* and *WRKY* familiy genes was suppressed or induced under water deficit stress for the genotypes, libraries (moderate and severe stress) and tissues (Figure 11). In general, the number of genes related to the *AP2/EREBP* family was higher than the *WRKY* family for both cultivars. When subjected to moderate and severe stress, leaves of Embrapa 48 presented more up-regulated genes (89 genes) in the *AP2/EREBP* family than BR 16 (33 genes) (Figure 11A). While for the *WRKY* classification, Embrapa 48 exhibited more negatively regulated genes than the sensitive cultivar (18 and 13, respectively). BR 16 presented 15 up-regulated genes in the moderate library, while Embrapa 48 showed 12 genes (Figure 11A). Thus, the differences between genotypes is more evident for the moderate stress and for the *AP2/EREBP* family.

The same pattern was detected for roots, in which the major contrast between the cultivars was found for the *AP2/EREBP* family (Figure 11B). Embrapa 48 activated 59 more genes and repressed 16 less genes when compared to BR 16. This discrepancy was not observed for the *WRKY* family; Embrapa 48 presented 45 up and 18 down-regulated genes compared to 27 and 13 for BR 16. In general, Embrapa 48 presented a more evident dynamic and positive expression of both gene families (Figure 11).

### Validation of gene expression

**Figure 12:**
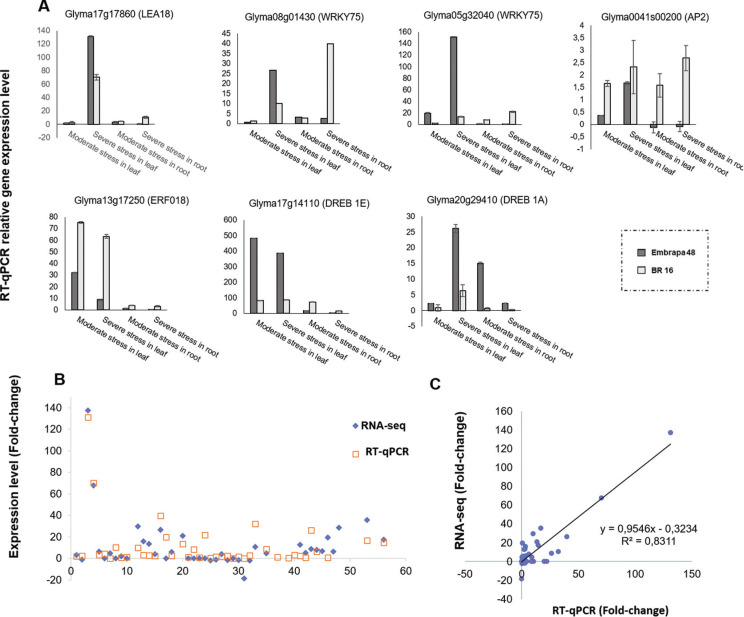
Correlation between RT-qPCR and RNA-seq analyses of the seven genes selected in soybean cultivars under water deficit conditions: Glyma17g17860 (*LEA 18*), Glyma08g01430 (*WRKY75*), Glyma05g32040 (*AP2*), Glyma0041s00200 (*AP2*), Glyma13g17250 (*ERF018*), Glyma17g14110 *(DREB1E),* and Glyma20g29410 (*DREB1A*). (A) RT-qPCR of the seven genes aforementioned. Gene expression level is relative to the control plants (0 min under stress). Fold-change (y-axis) and treatments to each gene (x-axis). (B) RNA-seq and RT-qPCR results for the seven genes validated in Embrapa 48 and BR 16 subjected to all stress treatments. (C) Correlation of the fold-change analyzed between RNA-seq (y-axis) and RT-qPCR (x-axis).

*LEA18*, which plays a crucial role in cellular dehydration tolerance, and some members of *AP2/EREBP* and *WRKY* families were selected for RT-qPCR analysis aimed at validating the results obtained in the RNA-seq libraries, as well as confirming that the water deficit stress treatment was successfully applied (Table 1 in S1). *LEA18* (Glyma17g17860) was highly up-regulated in leaf under severe stress, particularly for Embrapa 48, reaching nearly twice the value of fold-change (fc) (131.3) when compared with BR 16 (fc of 70.34) (Figure 12A). Glyma08g01430 (*WRKY75*) was highlighted in both tissues of plants subjected to severe stress. Considering leaf, Embrapa 48 obtained a fc of 26.6, while for BR 16 the fc was 9.94. For roots the fold-change was 39.79 and 2.52, respectively (Figure 12A). Glyma05g32040 (*AP2*) stood out in leaf from Embrapa 48 under severe stress (fc of 150.88), while BR 16 showed a fc of 13.54 (Figure 12A). Glyma0041s00200 (*AP2*) had a positively differential expression in BR 16 in all treatments. However, it was negatively regulated in root from Embrapa 48 in the two libraries (moderate and severe stress). For Glyma13g17250 (*ERF018*), the differential expression was more intense in leaf in moderate stress, reaching a fc of 32 and 75.25 for Embrapa 48 and BR 16 respectively, while under severe stress, the cultivars presented fcs of 8.8 and 63.19, in the same order (Figure 12A). Considering Glyma17g14110 (*DREB1E*) in leaf for both stress conditions, Embrapa 48 showed a more evident regulation (fc of 482.16 in moderate stress and 387 in severe stress) than BR 16 (fc of 82.74 in moderate stress and 86 in severe stress) (Figure 12A). Finally, Glyma20g29410 (*DREB1A*) was more intensively expressed in Embrapa 48, mainly in leaf of plants under severe stress, reaching a fc of 26.18, and in root in moderate stress with a fold-change of 15.05 (Figure 12A). The RNA-seq values showed low variation compared to the RT-qPCR analyses (Figure 12B). The expression pattern obtained was widely compatible between both assays (R^2^ = 0,8311) (Figure 12C). These results show that the drought treatments were applied successfully, besides validating the data obtained from the RNA-seq analysis.

## Discussion

Plants under water stress present physiological strategies, including biochemical and morphological modulations, as adaptive or defensive methods of coping with this stressful condition. A better understanding of the genetic mechanisms involved in these physiological, biochemical, and morphological responses to water deficit stress are crucial to define strategies for breeding programs, such as selecting superior parental genotypes or even developing transgenic or genomic edited lines aiming at drought tolerance. Therefore, the development of biotechnological tools for identification and characterization of promoters, genes, and other genetic factors that contribute to abiotic stress tolerance, such as drought, has great importance in the production of crops (Abdallah *et al.*, 2015; Cardi *et al.*, 2017).

This study examined the response of drought-tolerant and drought-sensitive soybean plants at a genetic level, identifying the molecular differences between the two types of cultivars. Here, the soybean genotypes demonstrated different molecular responses to conditions of water deficit, showing Embrapa 48 to be tolerant and BR 16 to be sensitive. Embrapa 48 showed a faster response to water deficit, presenting a greater number of DEGs since the first signs of stress. Embrapa 48 exhibited initial modulation of genes associated with drought, while maintained the level of the ones related to basic functions. Similar phenotyping was reported by Oya *et al.* (2004).

The *LEA18* gene was one of the genes selected to perform the validation of this study. LEA proteins are a large and highly diverse gene family present in plant species. LEAs have been supposed to play a role in various stress tolerance responses (Gao and Lan, 2016). Olvera-Carrillo *et al.* (2010) demonstrated that overexpression of a member of the LEA proteins confers tolerance to severe drought in *Arabidopsis thaliana*. It is suggested that LEA18 is not a membrane stabilizing protein, as observed for other proteins LEA. Instead, a possible function of LEA18 is the composition-dependent modulation of membrane stability, for example during signaling or vesicle-mediated transport (Hundertmark *et al.*, 2011). The up-regulation of the gene *LEA18* was more intense in Embrapa 48, demonstrating that, regarding drought tolerance responses, this cultivar is superior to BR 16.

According to the observations, especially in leaves, Embrapa 48 responds to water deficit stress quickly, presenting a higher number of up-regulated genes expressed in moderate stress library. This pattern persists throughout the gene classes, such as amino acid and sucrose related genes, as shown in Figure 5. In addition, both classes cited may be related to the osmoregulation mechanisms of the plant, as in the case of proline, trehalose, mannitol, ectoine, glycine and betaine, providing tolerance to cellular dehydration (Ha *et al.*, 2015). In summary, Embrapa 48 exhibits a faster initial gene modulation.

Therefore, increase in water deficit tolerance occurs mainly by osmotic adjustment and osmoprotective characteristics (Conde *et al.*, 2008). Proline is an amino acid that acts on the osmoregulation in plants under conditions of stress, conferring tolerance to cellular dehydration (Varshney *et al.*, 2011; Wani and Gosal, 2011). The accumulation of organic solute in response to drought is an important mechanism for maintaining cellular turgor, contributing to a reduction of water potential (Ψw), which increases the water absorption capacity of plants (Silva *et al.*, 2009). A higher concentration of genes involved in proline metabolism was found in Embrapa 48. Thus, osmoregulation is an efficient and positive tolerance response, delaying damage caused by low water content inside plant cells. Osmoregulation is considered one of the main and most important tolerance mechanisms, being able to define the degree of tolerance or susceptibility of a plant (Pantuwan *et al.*, 2002).

Sucrose has great metabolic importance in plants as well, being essential in tissues such as roots. According to studies conducted in maize (*Zea mays*) by Ogawa *et al.* (2005), sugars contributed to both initiation and elongation of roots, collaborating for a better performance of the plant in drought conditions. Moreover, auxin and sugars have been found to play an important role in the initiation of lateral roots (Takahashi *et al.*, 2003). These results corroborate the data obtained in this study, since a predominance of sucrose-related genes was detected among the up-regulated genes in leaf and root samples from Embrapa 48 subjected to moderate water deficit (Figure 5). Furthermore, the lack of these genes being up-regulated in BR 16 evidences its sensitivity to drought (Figures 5 and 8).

Gargallo-Garriga *et al.* (2014) suggested that leaves and roots rely on a contrasting metabolism to respond to changes in environmental conditions, presenting different physiological mechanisms and functions in stress adaption. Many metabolic products and soluble sugars are produced in leaves but are allocated and used in roots due to root energy requirements for the assimilation of soil resources and growth. However, other molecules, such as terpenes and metabolites related to anti-stress mechanisms, are increased in leaves under drought. Thus, it is possible that the higher sucrose levels in leaves from Embrapa 48 under moderate stress provided energy to primary metabolism and defense responses, inducing osmoregulation (Figure 5). Furthermore, solutes are accumulated to prepare cells for translocation of metabolites from leaves to roots. On the other hand, BR 16 only showed predominance of up-regulated genes in root samples under severe stress (Figures 8 and 9). The metabolism of roots is more strongly controlled by homeostasis and conserved compared to leaves, as also suggested by Gargallo-Garriga *et al.* (2014).

The reduction of stomatal conductance is a means of defense against cell dehydration. Rodrigues *et al.* (2012) developed tests similar to the ones performed in the present study and demonstrated superior stomatal conductance in Embrapa 48 compared to BR 16 for all levels of water deficit. Thus, BR 16 has probably developed conditions of oxidative stress prematurely due to stomatal closure and consequent reduction of CO_2_ assimilation. This leads to accumulation of ATP, energy reduction (NADPH), and reduction of the final acceptor of the electron transport chain (NADP^+^). The excessively reduced activity of NADPH can induce a strong limitation of the electron transport chain. In this process, electrons can escape and react with molecular oxygen (O_2_), forming reactive oxygen species (ROS) (Cavatte *et al.*, 2011). This disordered accumulation of ROS causes oxidative stress.

Down-regulation of the TCA cycle and amino acid biosynthesis apparently acts to prevent energy loss under conditions of oxidative stress (Urbanczyk-Wochniak *et al.*, 2003). In this context, BR 16 was possibly under conditions of oxidative stress since the first level of stress, presenting a higher number of down-regulated genes, whereas Embrapa 48 only showed a down-regulated gene profile in the severe stress library. Moreover, the high level of up-regulated peroxidases found in leaves of Embrapa 48 with moderate stress is another characteristic that reinforces that hypothesis. Peroxidases act by combating ROS, thus, it can be inferred that Embrapa 48 did not succumb to oxidative stress (Figure 7).

Additionally, it is evident that the predominance of up-regulated activity of ABA-related genes in Embrapa 48 (Figure 7) has led to a whole response, activating signaling molecules of stress, such as ROS, and at the same time recruiting the synthesis of peroxidases (Figure 7), therefore, providing equilibrium to the system. Jiang and Zhang (2002) reported that a raise in ABA content precedes ROS increase, followed by higher activity of antioxidant enzymes. Yamaguchi-Shinozaki and Shinozaki (2006) also concluded that water deficit stress induces the accumulation of ABA in plants, which promotes changes in gene expression and in stomata closing, leading to concomitant reduction in transpiration, in carbon assimilation and water loss.

Interestingly, Embrapa 48 is able to preserve primary metabolism genes activated, which act upon basic function, such as plant development, light reactions, photorespiration equilibrium, and energy supplement (Figures 5, 6, and 7), concomitantly, it maintains genes related to the secondary metabolism and stimulates the expression of specific genes to combat the effects of stress (Figure 7).

Among the twenty differentially expressed genes in leaf samples from Embrapa 48 subjected to moderate and severe stress, the genes that excelled in expression patterns exclusively in these two treatments, were annotated in several classes: histone H2A variant 1-related, histone H3, glutathione s-transferase u1-related, histone H3, histone H4, histone H2B, raffinose synthase, proline-rich protein 4, protein kinase domain (Pkinase), zinc finger protein-related, 3, 4-dihydroxy-2-butanone-4-phosphate synthase, and G-box-binding factor 2-related. Some of these categories are linked to the processes of osmoregulation, energy balance of cells and gene regulation.

Several of the pathways described above as active or affected by water deficit were previously reported in soybean in a transcriptome study conducted by Rodrigues *et al.* (2015), showing the impact of drought on the plant metabolism. However, their focus was the interactions between water deficit and the circadian cycle in soybean cultivars susceptible to water deficit, as the case of the investigation performed by Marcolino-Gomes *et al.* (2015). In the present study, the comparison with a drought tolerant material adds new information about which are the mechanisms involved in the greater tolerance to this abiotic stress.

The better performance of Embrapa 48 under water deficit is supported by the strong presence of up-regulated genes related to TFs and drought (Figure 7). Stress-induced transcription factors are considered powerful targets, being a natural starting point for mechanisms regulating the expression of several genes, and key to genetic transformation strategies (Woodrow *et al.*, 2012).

*AP2/EREBP* and *WRKY* are among the numerous gene families activated during the stress stages in different tissues. These genes are described as central TFs in water deficit tolerance and other stresses in several plant species. The increase of tolerance to such stresses (salinity, ionic stress, drought, and low temperature) were obtained in many transgenic plants of different species utilizing the TF DREB, such as *Oryza sativa* (Paul *et al.*, 2015), *A. thaliana* (Chen *et al.*, 2015) *Saccharum spp. Hybrid. Co 86032* (Augustine *et al.*, 2015), *Glycine max* (Rolla *et al.*, 2014), or the TF *WRKY* in *Nicotiana tabacum* (Sun *et al.*, 2015) and *Oryza sativa* (Cai *et al.*, 2014).

Between these two large families, the *AP2/EREBP* stands out. According to the expression standards presented here, it has a greater number of genes activated in the tolerant cultivar throughout the stress periods (Figure 11). This may be an indication of a greater and direct participation of the *AP2/EREBP* family in drought tolerance responses compared to *WRKY*. The expression of the *GmDREB1* gene, an *AP2/EREBP* member, under various abiotic stress conditions in soybean had been examined. *DREB1E*, for instance, responded to heat (42 °C), cold (4 °C), NaCl (250 mM), and drought (four and seven days) stress conditions (Kidokoro *et al.*, 2015). The RT-qPCR data confirm the differences in the pattern of regulation of these genes between the tolerant and sensitive genotypes, and in many cases these genes were more strongly induced in the tolerant cultivar (Figure 12A). For instance, it was observed that *DREB1E* (Glyma17g14110) was highly active in leaf of Embrapa 48 under moderate and severe stress, participating in the water deficit stress tolerance process (Figure 12A). Finally, the results from the RT-qPCR validates the data obtained from the RNA-seq analysis (Figure 12B and C).

## Final considerations

This study compared the different functions and metabolic activity of differentially expressed genes in leaves and roots of two soybean genotypes under water deficit conditions. The difference found in the response of Embrapa 48 and BR 16 in leaf and root samples is remarkable and explains the better performance of the Embrapa 48 cultivar under drought conditions. In fact, the leaves generated a greater number of up-regulated genes, and data showed that Embrapa 48 responds to water deficit faster than BR 16, presenting a larger number of DEGs since the first signs of drought (moderate level). Furthermore, the genes identified in our study may be used as potential candidates for future investigations aimed at drought tolerance in soybean, since these genes were exclusive to the tolerant cultivar, or presented high levels of differential expression, probably playing an important role in the tolerance response. Finally, the *AP2/EREBP* and *WRKY* genes selected in this work might be potential study tools in the analysis of their promoters and regulatory mechanisms.

## Supplementary Material

The following online material is available for this article:

Table S1 - Name and sequence of the primers designed for the seven genes selected for real-time PCR analysis.
