# Supplementary material for: Differential gene expression in response to water deficit in leaf and root tissues of soybean genotypes with contrasting tolerance profiles
Source: Genet Mol Biol. 2020 May 29;43(2):e20180290. doi: 10.1590/1678-4685-GMB-2018-0290 (PMC7263426; doi:10.1590/1678-4685-GMB-2018-0290)
Supplement: Supplementary file 1 [file 1415-4757-GMB-43-2-e20180290-s1.pdf]

Genetics and Molecular Biology 43, 2 (2020)

Supplementary Material to “Differential gene expression in response to water deficit in leaf and root tissues of soybean genotypes with contrasting tolerance profiles”

Table S1 – Name and sequence of the primers designed for the seven genes selected for real-time PCR analysis.

Primer name- Wm82.a1.v1	Wm82.a2.v1	Sequence
Glyma17g17860-F	Glyma.17g164200	5´AAAGGCACAGAGTGATGAAT3´
Glyma17g17870-R		3´CTTGATGACCTTGTGTACCA5´
Glyma08g01430-F	Glyma.08g11300	5´CCTGAAAGGTGGCAAAGAAA3´
Glyma08g01430-R		3´CTTCACATTGCAACCTCGAT5´
Glyma05g32040-F	Glyma.05g186700	5´GGAGGAAGAACCGAGGAGAA3´
Glyma05g32040-R		3´GCTTTGTTGCCTCTGAAACG5´
Glyma0041s00200-F	Glyma.U037700	5´GCTTGGACAGAAGCAACCTG3´
Glyma0041s00200-R		3´TTCCTTTTTGCCCTTCCTT5´
Glyma20g29410-F	Glyma.20g155100	5´AGGTCAGCCTGCCTCAACTT3´
Glyma20g29410-R		3´GGCACCATCCCTTCTTCTTG5´
Glyma13g17250-F	Glyma.13g112400	5´GAAAATGGGGCAAATGGGTA3´
Glyma13g17250-R		3´GTCGGGGAAATTGAACTTGG5´
Glyma17g14110-F	Glyma.17g131900	5´AGGATTTGGCTCGGAACGTA3´
Glyma17g14110-R		3´CAATCAGCAACGGCATCAAT5'
